# Supplementary material for: Distribution of soil microorganisms in different complex soil layers in Mu Us sandy land
Source: PLoS One. 2023 Apr 7;18(4):e0283341. doi: 10.1371/journal.pone.0283341 (PMC10081775; doi:10.1371/journal.pone.0283341)
Supplement: Attachment — Submitted filename: Response to Reviewers.docx [file pone.0283341.s002.docx]

**Response to Reviewers**

1. Please ensure that your manuscript meets PLOS ONE's style requirements, including those for file naming. The PLOS ONE style templates can be found at
[https://journals.plos.org/plosone/s/file?id=wjVg/PLOSOne_formatting_sample_main_body.pdf](https://journals.plos.org/plosone/s/file?id=wjVg/PLOSOne_formatting_sample_main_body.pdf) and
[https://journals.plos.org/plosone/s/file?id=ba62/PLOSOne_formatting_sample_title_authors_affiliations.pdf.](https://journals.plos.org/plosone/s/file?id=ba62/PLOSOne_formatting_sample_title_authors_affiliations.pdf.)

**Response**: The author has modified according to the journal template requirements.

2. We noticed you have some minor occurrence of overlapping text with the following previous publication(s), which needs to be addressed:

[https://peerj.com/articles/13561/](https://peerj.com/articles/13561/)

In your revision ensure you cite all your sources (including your own works), and quote or rephrase any duplicated text outside the methods section. Further consideration is dependent on these concerns being addressed.

**Response**: The author rewrote the repeated text. References are cited. Thank you.

3. PLOS requires an ORCID iD for the corresponding author in Editorial Manager on papers submitted after December 6th, 2016. Please ensure that you have an ORCID iD and that it is validated in Editorial Manager. To do this, go to ‘Update my Information’ (in the upper left-hand corner of the main menu), and click on the Fetch/Validate link next to the ORCID field. This will take you to the ORCID site and allow you to create a new iD or authenticate a pre-existing iD in Editorial Manager. Please see the following video for instructions on linking an ORCID iD to your Editorial Manager account: [https://www.youtube.com/watch?v=_xcclfuvtxQ.](https://www.youtube.com/watch?v=_xcclfuvtxQ.)

**Response**: The author provided ORID iD: 0000-0003-3383-6341

**Reviewer #1: Author are recoomended to improve manuscript**
1."The soft rock in Mu Us Sandy Land has rich resources and high content of clay minerals" In abstract pl write full form of Mu Us .

**Response**: "Mu Us Sandy Land" is an official name that refers to a specific place, and therefore does not exist in the full form of Mu Us.

2."The four volume ratios of soft rock to sand were respectively 0:1 (CK), 1:5 (P1), 1:2 (P2) and 1:1 (P3)."In abstract pl explain about CK,P1,P2.P3

**Response**: The four volume ratios of soft rock to sand were respectively 0:1, 1:5, 1:2 and 1:1. And CK, P1, P2 and P3 were used to represent the above four volume ratios in turn.

3.I strogly recommend for publication of this work.soil parameter with meta genomics provide insight about soils are remarkable

**Response**: Thank you very much for your suggestion. Other questions have been modified.

4.Author are strongly suggested to pl write full farm of abbreviation at least once in manuscript so that reader feel easy.

**Response**: The author gives a complete explanation where the abbreviations first appear in the whole text. In addition, the author of each part makes a complete description of abbreviations. Thank you！

5.Author are suggested to write future prospective of this work in conclusion.How this work might be helpful to increase diversity, conservation and crop production.

**Response**: The author has added the future prospective of this work in conclusion. “The improvement of comprehensive properties of aeolian sandy soil will provide a good theoretical basis for the development of green agriculture and carbon emission reduction effect in the next step. Therefore, in the future, the author will continue to study the function and metabolism of microorganisms in sandy land, and carry out the isolation and identification of relevant carbon-fixing microorganisms.”

**Reviewer #2:**

1. The study was well-structured, yet some areas of the manuscript need attention.

**Response**: The author revised the full text according to the comments. Thank you.

2. Please correct the grammatical errors throughout the manuscript. Write in passive voice (Line no. 169 & Line no.173)

**Response**: The author has modified the sentence in the passive voice.

3. It will be Phylum Actinobacteria. The word Phylum must be written first, then the name of the Phylum.

**Response**: The author has revised the whole text. Thank you.

4. There were observations but adequate discussions were missing.

**Response**: The author adds a detailed discussion to the research results. Details are as follows:

Kang *et al*. [26] showed that the spatial structure of soil and the thickness of different soil layers had significant effects on soil nutrients, which was similar to the results of this study.

Among many wetlands, Phylum *Proteobacteria* has the highest relative abundance because of their strong adaptability to the environment [28]. The Phylum *Actinobaciota* has the highest abundance in this study, followed by Phylum *Proteobacteria*, indicating that Phylum *Proteobacteria* has high abundance in both dry land and wetland.

Moreover, the increase of diversity in the surface layer was greater than that in the bottom layer. It may be due to the addition of soft rock clay minerals in the surface soil, which was greatly affected by the soil parent material [31].

Bacteria have the highest diversity and the most stable community structure in medium-alkaline soil, but small changes in pH value may lead to the formation of different community structures [32]. In this study, the pH values of P1 and P3 in 0-30 cm soil layer were basically the same, and the pH values of P1 and P2 in 30-60 cm soil layer were basically the same, showing the similarity of community structure.

……

5. Explain the observed relationships between soil properties and the bacterial abundances from previous literatures.

**Response**: The author has added. “However, the study of Kong et al. [26] was inconsistent with the results of this paper, arguing that pH was the main reason for affecting the bacterial community structure of surface soil, and ammonium nitrogen was the main reason for affecting the bacterial community of deep soil. This was because deep soil was more stable, while surface soil was susceptible to temperature, humidity and human activity.”

6. The significance of the study results was not reflected in the discussions/conclusions.

**Response**: The authors add research significance to the conclusion. “The results of this study provide practical significance for the reclamation of sandy land and the increase of cultivated land resources.”
